# Redirecting tumor-associated macrophages to become tumoricidal effectors as a novel strategy for cancer therapy

**DOI:** 10.18632/oncotarget.17061

**Published:** 2017-04-12

**Authors:** Xiang Zheng, Kati Turkowski, Javier Mora, Bernhard Brüne, Werner Seeger, Andreas Weigert, Rajkumar Savai

**Affiliations:** ^1^ Max Planck Institute for Heart and Lung Research, Department of Lung Development and Remodeling, Member of the German Center for Lung Research (DZL), Bad Nauheim, Germany; ^2^ Institute of Biochemistry I, Goethe-University Frankfurt, Frankfurt, Germany; ^3^ Department of Internal Medicine, Universities of Giessen and Marburg Lung Center (UGMLC), Member of the DZL, Justus Liebig University, Giessen, Germany

**Keywords:** tumor microenvironment, macrophages, repolarization, cancer progression, metastasis

## Abstract

Cancer research in recent decades has highlighted the potential influence of the tumor microenvironment on the progression and metastasis of most known cancer types. Within the established microenvironment, tumor-associated macrophages (TAMs) are one of the most abundant and crucial non-neoplastic cell types. The polarization of macrophages into tumor-suppressive M1 or tumor-promoting M2 types is a fundamental event in the establishment of the tumor microenvironment. Although ample evidence indicates that TAMs are primarily M2 polarized, the mechanisms responsible for the regulation and maintenance of M1 and M2 polarization imbalance remain unclear. The manipulation of this critical axis through three main approaches may provide new strategies for cancer therapy — (I) specific interference with M2-like TAM survival or inhibiting their signaling cascades, (II) repression of macrophage recruitment to tumors, and (III) repolarization of tumor-promoting M2-like TAMs to a tumoricidal M1-like phenotype. This review summarizes current strategies for cancer intervention via manipulation of macrophage polarization, with particular focus on composition of the tumor microenvironment and its influence on cancer progression and metastasis. It is clear that additional fundamental and preclinical research is required to confirm the efficacy and practicality of this novel and promising strategy for treating cancer.

## THE TUMOR MICROENVIRONMENT AND TUMOR-ASSOCIATED INFLAMMATION

Numerous cancer risk factors can be linked to chronic inflammation which was recently recognized as a hallmark of cancer [1]. The physiological microenvironment of any given organ is usually tumor-suppressive, yet the microenvironment is vulnerable to chronic inflammation caused by, for example, microbial infection or triggers that induce sterile inflammation. As a result, a tumor-promoting microenvironment (TME) can be established. Generated by tumor cells and surrounding stroma, the TME is composed of vascular and lymphatic endothelial cells, pericytes, fibroblasts, immune cells, an altered extracellular matrix (ECM) and is in early stages restricted by a basement membrane [2]. The TME has a fundamental role in tumor progression, metastasis and immunosuppression, and it also accounts for the resistance of tumor cells to drug treatment [2]. Therefore, remodeling of the TME provides novel and promising opportunities for cancer therapy.

**Figure 1:**
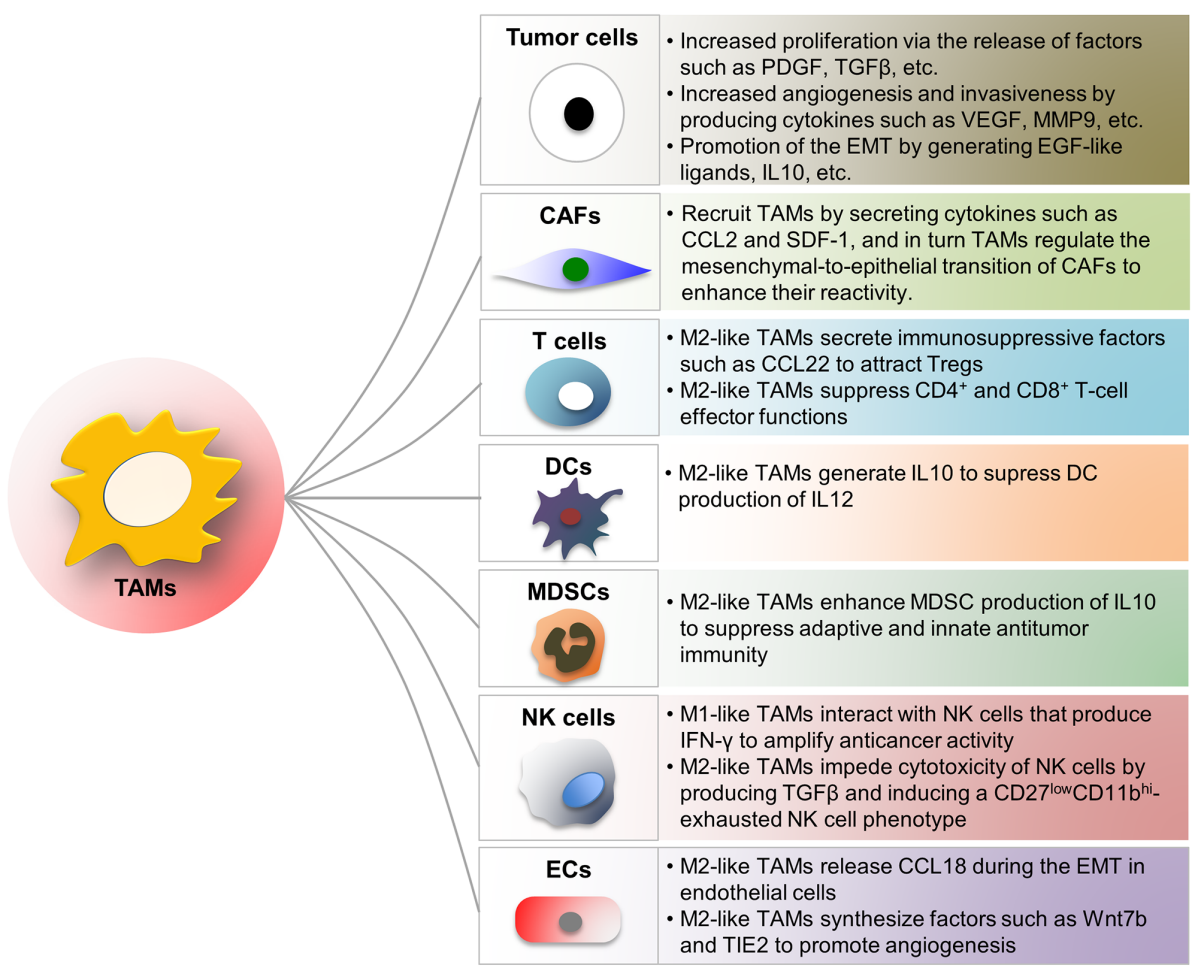
Influence of TAMs on other cells in TME TAMs interact with tumor cells and other tumor-infiltrating immune cells to influence tumor angiogenesis, invasion as well as metastasis. Some of the interactions mentioned in this review are depicted in the figure. TAM, tumor-associated macrophage; CAF, cancer-associated fibroblast; MDSC, myeloid-derived suppressor cell; NK, natural killer cell; DC, dendritic cell; Treg, regulatory T cell; EC, endothelial cell.

As the cancer progresses, normal fibroblasts are converted into cancer-associated fibroblasts (CAFs) that continuously release growth factors such as TGFβ that can regulate the epithelial–mesenchymal transition (EMT) [3, 4]. CAFs are the most prominent cell type within the tumor stroma, and are divided into several subpopulations based on their derivation and marker expression. CAFs acquire the features of myofibroblasts, including increased production of α-smooth muscle actin (α-SMA), whereupon they facilitate tumor initiation and progression [3]. In addition to TGFβ, CAFs release stromal cell-derived factor 1 (SDF-1/CXCL12), which recruits endothelial progenitor cells to the tumor site to facilitate angiogenesis and directly promote tumor growth via binding to its cognate receptor, CXCR4, expressed by cancer cells [3]. Releasing cytokines and chemokines to attract and regulate innate and adaptive immune cells is a dominant mechanism by which CAFs modulate cancer-related inflammation. For instance, CAFs secrete CC chemokine ligand 2 (CCL2), which recruits macrophages to the tumor site through binding to its receptor CCR2 [4]. Moreover, CAFs drive SDF-1/CXCL12 production, which is also a chemoattractant of macrophages and promote M2 macrophage polarization in prostate cancer. In turn, M2 macrophages regulate mesenchymal-to-epithelial transition (MET) of fibroblasts, leading to their enhanced reactivity (Figure 1) [5]. Aside from cytokine and chemokine secretion, modulation of the ECM by CAFs also promotes the enrichment of macrophages. Hyaluronan is a major component of the ECM, and tumor-associated macrophages (TAMs) are preferably attracted to hyaluronan-rich stromal areas [6]. Depletion of hyaluronan synthase 2 in CAFs reduces TAM recruitment and thereby attenuating tumor angiogenesis and lymphangiogenesis [6]. In addition, Martinez-Outschoorn et al. suggested an “autophagic tumor stroma model of cancer metabolism” as a mechanism for the tumor-promoting effect of CAFs [7]. Specifically, they propose that tumor cells induce hypoxia-inducible factor 1α (HIF-1α) and nuclear factor κB (NF-κB) in CAFs and drive autophagy in CAFs, leading to nutrient release to support tumor cell metabolism.

The main populations of tumor-promoting inflam-matory cells are TAMs, TIE2-expressing monocytes (TEMs), tumor-associated neutrophils (TANs), myeloid-derived suppressor cells (MDSCs), natural killer (NK) cells, mast cells (MCs), dendritic cells (DCs) and T cells. TAMs are among the most versatile tumor-infiltrating inflammatory cells and principally originate from hematopoietic bone marrow precursors [8]. Classically activated (M1) macrophages and alternatively activated (M2) macrophages are two distinct states of polarized macrophages driven by cytokine repertoire of T helper cells (Th1 and Th2 respectively). TAMs that may represent up to 50% of the tumor mass are mainly M2-like in invasive cancers and support virtually all hallmarks of cancer by generating numerous growth factors, cytokines and ECM-remodeling molecules such as CCL2, CXCL12, CXCR4, TGFβ, VEGF, PDGF, COX-2 and metalloproteinases to regulate tumor growth, migration as well as angiogenesis [8–11]. TAMs dynamically interact with T cells during tumor progression. M1-like TAMs direct T cells towards Th1 tumoricidal responses, whereas immunosuppressive factors such as CCL22 secreted by M2-like TAMs suppress CD4^+^ and CD8^+^ T cells effector functions and recruit regulatory T cells (Tregs) to the TME [8, 12–14]. In addition to directing T cell responses, M1-like TAMs also interact with NK cells that produce IFN-γ to amplify anti-tumor activity [15]. In contrast, cytotoxicity of NK cells is impeded by M2-like TAMs through producing TGFβ and inducing CD27^low^CD11b^hi^-exhausted NK cell phenotype (Figure 1) [16]. The mechanisms involved in the function and polarization of TAMs during tumor progression will be further discussed in the following sections.

MDSCs in mice consist of two main subtypes - CD11b^hi^ Ly6G^hi^ Ly6C^low^ CD49d^low^ granulocytic-MDSCs and CD11b^hi^ Ly6G^low^ Ly6C^hi^ CD49d^hi^ monocytic-MDSCs. Both subtypes are derived from immature bone marrow progenitors that are mobilized by a number of tumor-associated inflammatory factors, and their relative proportions depend on the tumor type and organism [17, 18]. MDSCs greatly influence the immunosuppressive effects of the TME by impairing CD8^+^ T cell and NK cell functions. They release limited amounts of nitric oxide by expressing both inducible nitric oxide synthase (iNOS) and arginase 1 and induce the differentiation of Tregs that maintain an immunosuppressive environment by secreting TGFβ and interleukin 10 (IL10) and competitively binding and neutralizing the anti-tumor cytokines such as IL2, IL7, IL12 and IL15 [17, 18]. TAMs enhance MDSC production of IL10, depend on which macrophage production of IL12 is reduced [19]. Hence, MDSCs are impediment of effective immunotherapy and their reduction may facilitate immunosurveillance to suppress tumor progression (Figure 1) [19].

Neutrophils make up 50–70% of circulating leukocytes. Increasing immunohistochemical evidence has shown that an elevated number of TANs indicates a poor prognosis in colon carcinoma, bronchioloalveolar carcinoma, gastric carcinoma, renal carcinoma and melanoma [20]. Similar to TAMs, TANs can be categorized intotumor-suppressive (N1) and tumor-promoting (N2) subtypes. Again, TGFβ plays an important role, because depletion of TGFβ drives conversion of TANs to the N1 state, and its overproduction prevents this conversion [21]. TANs, which are derived from the circulation, are recruited to the tumor site through TME-generated chemokines binding to CXCR1 and CXCR2. Once in tumors, TANs release factors such as oncostatin M that induce tumor cells to produce VEGF and matrix metallopeptidase 9 (MMP9) to facilitate angiogenesis [22]. Although activated neutrophils which secrete IL8 and TNFα recruit macrophages to the site of inflammation, it remains unknown whether the interaction between TANs and TAMs in the TME is similar to that in non-tumoral chronic inflammatory environment.

MCs are granulocytic immune cells that play multifaceted roles in tumor progression and inhibition. The multifaceted feature of MCs is due to plastic potential to generate pro- or anti-tumor subtypes in response to specific TME stimuli [23]. Histamine produced by MCs polarizes CD4^+^ T Cells toward a Th2 phenotype that favors tumor development through histamine receptor type 2 (H2R). In addition, histamine recruits Tregs to establish an immunosuppressive microenvironment [23]. Furthermore, MCs recruit TAMs to promote tumor invasion via activated PI3K/AKT pathway in inflammation-induced colon cancer [24]. Hence, MCs contribute to mold the TME by interacting with other tumor-infiltrating immune cells, which engenders the opportunity to develop MC-targeted therapies for cancer patients.

DCs are professional antigen-presenting cells. Conventionally, intracellular antigens, such as viral proteins, are presented on MHCI molecules to CD8^+^ T cells, whereas extracellular antigens, such as bacteria and toxins, are presented on MHCII molecules to CD4^+^ T cells. However, DCs have the ability to cross-present extracellular antigens to CD8^+^ T cells, which is important for tumor-suppressive immunity. The mechanism by which the TME inhibits the ability of DCs to present antigens effectively is to retain DCs in an immature state, which blocks expression of co-stimulatory molecules, resulting in tolerance through T cell deletion [25]. Additionally, TAM-derived IL10 inhibits the production of IL12 by dendritic cells, ultimately leading to suppressed CD8^+^ T cell responses and DC tumor-suppressive functions (Figure 1) [26].

## TUMOR-ASSOCIATED MACROPHAGES

### Macrophage development

Macrophages play important roles in shaping tissues during embryogenesis. They appear from embryonic day 8 (E8) in mice and are involved in branching morphogenesis, the generation of adipose tissue and vascular patterning [27]. In the embryo, the earliest macrophages are derived from mesenchymal progenitors in the yolk sac. Subsequently, they migrate into embryonic tissues as soon as a functional vasculature is established. Accumulating studies indicate that yolk sac–derived macrophages are long-lived, self-sustaining cells [27]. A second wave of tissue macrophages is derived from erythro-myeloid progenitors (EMPs) that colonize the fetal liver at approximately E9. EMPs differentiate into pre-macrophages and subsequently colonize embryonic tissues to differentiate into tissue-specific macrophages. These EMP-derived macrophages are again long-lived and self-sustaining [28]. Hematopoiesis in bone marrow starts after birth, generating bone marrow–derived monocytes as a third wave of macrophage progenitors. In contrast to embryonic macrophages, bone marrow–derived macrophages are usually short-lived, rarely proliferate and are continuously replaced [27, 29]. Therefore, a mixture of macrophages arising from different progenitors during ontogeny could be expected in adult tissues. However, the tissue macrophage pool in adult organs shows some degree of specificity. For example, yolk sac macrophages constitute the vast majority of microglia in the central nervous system owing to establishment of the blood–brain barrier during embryogenesis, which precludes the influx of fetal or adult monocytes [27]. In other tissues, yolk sac macrophages are replaced by fetal EMP-derived or adult monocyte-derived macrophages to some extent [28]. For instance, adult epidermal macrophages, Langerhans cells and alveolar macrophages are derived from EMP-dependent macrophages that proliferate locally, whereas dermal macrophages and intestinal macrophages are constantly replenished by adult monocytes and do not proliferate *in situ*. Furthermore, tissue macrophage origins change if the tissue is subjected to inflammation because inflammatory monocytes are recruited to the inflamed areas from the circulation and differentiate into macrophages [27, 29]. As for the origin of TAMs, a study using primary mouse mammary tumor suggests that most of TAMs arise from the circulating Ly6C^hi^CCR2^hi^ monocytes derived from bone marrow hematopoietic stem cells [8]. Moreover, proliferation of resident macrophages and *in situ* monocyte-macrophage differentiation are the other origins of TAMs [30], and photoconvertible fluorescent lineage tracing of spleen indicates splenic monocytes are a minor source of TAMs [31]. Thus, both the original macrophage pool of a tissue and adult monocytes might contribute to the pool of TAMs in cancer [8]. However, local TME, shaped by a varying content of cytokines, growth factors and oxygen, as well as the presence of tumor cells, rather than ontogeny, appear to contribute to TAM function [32, 33].

### Macrophage heterogeneity

Macrophages are innate immune cells that specialize in maintaining tissue homeostasis. They command a broad sensory arsenal to detect perturbations in tissue integrity and possess a remarkable functional plasticity to combat diseases [27]. Macrophages reside in distinct tissues, including the liver (Kupffer cells, which are involved in iron storage, steatosis and liver repair), lungs (alveolar macrophages, which contribute to clearance of particulates), brain (microglia, which play a role in the removal of naturally aging neurons), skin (Langerhans cells, which are involved in antimicrobial immunity and skin immunosurveillance), spleen (splenic macrophages, which assist in the transport of microbial antigens to B and T cells and clear aged red blood cells) and other tissues, such as the gastrointestinal tract, cardiovascular system and granulomata [29]. That macrophages possess specialized functions in distinct anatomical locations underscores their heterogeneity.

The lineage-determining transcription factor for macrophages is PU.1, which determines the availability of factors necessary to generate the vast spectrum of different tissue macrophages [33]. Other stimulus-specific transcription factors include myocyte-specific enhancer factor 2c and SMAD in microglia, PPARγ in alveolar macrophages, PU.1-related factor (SPI-C) in iron-recycling macrophages of the spleen and bone marrow and GATA binding protein 6 (GATA6) in peritoneal macrophages [33]. These examples illustrate that the tissue microenvironment likely dictates the genetic signature of its resident macrophages by inducing expression of specific transcription factors.

**Figure 2:**
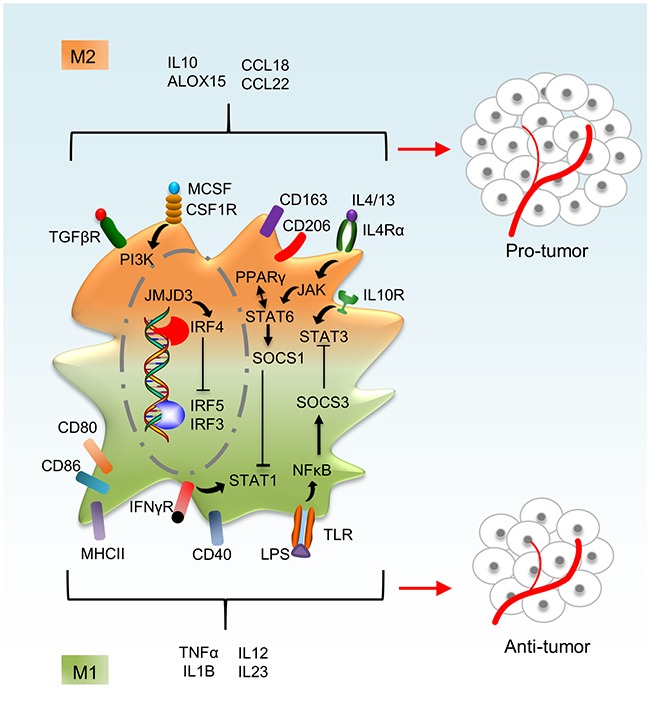
Macrophage activation phenotypes Macrophages are activated either classically (M1 phenotype) or alternatively (M2 phenotype). M2-polarized macrophages express high levels of CD206, CD163 and TGFβR, whereas M1 macrophages express high levels of CD40, CD80 and CD86 on the cell surface. STAT1 and STAT3 are highly activated in M1 phenotype and STAT6 in M2 phenotype. IRF3, 5 and 7 are activated in M1 phenotype, whereas IRF4 is activated in M2 phenotype. High levels of the cytokines and chemokines such as TNFα, IL1B and IL12 are observed in M1 phenotype and factors such as IL10, ALOX15 and CCL18 are highly expressed in M2 phenotype.

Independent of genetic imprinting owing to ontogeny or differentiation in a specific steady-state microenvironment, macrophages need to retain a high level of functional plasticity to respond to inflammatory stimuli of varying nature [32, 33]. Indeed, a plethora of macrophage phenotypes can be induced by different stimuli or by the same stimulus at different concentrations or different exposure times [34]. Following early observations of macrophage heterogeneity, two discrete activation states of macrophages were identified (Figure 2). Macrophage activation by activated Th1 cell–derived IFN-γ in combination with TNFα or the activation of toll-like receptors (TLRs) by bacterial cell wall components such as lipopolysaccharides (LPS) creates cells with a strong pro-inflammatory profile [35]. IFN-γ–stimulated macrophages show a transcription factor signature characterized bysignal transducer and activator of transcription 1 (STAT1) and interferon regulatory factor 3 (IRF3) [34, 35]. These transcription factors enable ‘classically activated’ M1 macrophages to generate pro-inflammatory mediators such as TNFα, IL1B, IL12, IL23 and reactive oxygen and nitrogen species and to present antigens to T cells via induction of MHCII molecules [32, 35]. M1 macrophages are potent defenders against microbes and are able to eliminate tumor cells. In contrast, macrophage activation by activated Th2 cell–derived IL4 or IL13 produces an alternative set of cytokines and chemokines that oppose the repertoire of classically activated M1 macrophages, and these ‘alternatively activated’ macrophages are designated as M2 macrophages. In addition to expressing phagocytic receptors such as the mannose receptor (CD206), M2 macrophages also produce the ECM components and growth factors to promote tissue remodeling and combat extracellular parasites [35], and their transcription factor profile is dominated by STAT6 and IRF4 [35]. Although the M1 and M2 macrophage distinctions are helpful for investigation, they hardly do justice to the multitude of macrophage phenotypes that are observed in tissues. Moreover, macrophage activation states are more transient than the stable M1/M2 activated macrophages, which maintain functional flexibility. Macrophage responses to any stimulus change over time and usually revert to the original state, and M2 macrophages readily acquire even more potent M1-associated functions when they are subsequently stimulated with TLR ligands or IFN-γ [32, 36]. The ability to switch phenotypes enables macrophages to perform different tasks sequentially during the course of an inflammatory reaction, including pathogen killing, engulfing and digesting cellular debris, stimulating adaptive immunity and promoting tissue regeneration [32, 33, 35].

**Table 1:** Clinical and experimental therapeutic approaches targeting TAMs

Mechanism of intervention	Target	Strategy	Reference
Interference with TAM survival	Legumain	Legumain-based DNA vaccine	[13]
	CD204	Anti-204 immunotoxin	[61]
	IL4Rα/CD124	RNA aptamer	[14]
	CD52	Alemtuzumab^▲^	[100]
	FRβ	Anti-FRβ mAb	[63]
	Cytotoxicity in monocytes	Trabectedin (ET-743) ^▲^	[58–60]
		Liposomal clodronate	
		M2pep	
Inhibition of macrophage recruitment	CCL2/CCR2	Neutralizing antibody CNTO 888	[49–52, 69]
		CCL2 inhibitor bindarit	
		CCR2 kinase antagonist PF-04136309^▲^	
		Luteolin	
	CSF1/CSF1R	Neutralizing antibody RG7155	[64–67]
		CSF-1R inhibitor PLX6134, GW2580 or PLX3397	
		Liposomal bisphosphonate	
		miR-26a	
Repolarization of M2-like TAMs towards an M1-like phenotype	CSF1/CSF1R	CSF-1R inhibitor BLZ945	[53]
	Microenvironmental stimuli	IL12	[36, 48, 71–75]
		IFN-γ	
		polyl:C	
		bacteria-mediated tumor therapy	
	Vascular normalization	Zoledronic acid^▲^	[76–79]
		Histidine-rich glycoprotein	
		Hydrazinocurcumin	
		DMXAA^▲^	
	NF-κB pathway	TLR agonists (polyl:C, CpG-ODN, TLR9 ligand, IL10R mAb)	[12, 81–84, 101]
		PA-MSHA	
		Flavone glycoside Baicalin	
		CD40 mAb	
		Natural compound corosolic acid	
	MAPK/ERK pathway	CuNG	[85]
	Epigenetic regulation	Overexpressing miR-155/miR-511-3P	[87–89, 102]
		Deletion of miR-146a	
Nanoparticle and liposome-based drug delivery systems	Engulfed by TAMs and subsequently target cancer cells	Mitoxantrone-loaded SLNsCisplatin-and cyclodextrin-loaded polymer nanoparticlesAlbumin nanoparticle–based Abraxane ▲Liposomal Doxil	[92, 93]

^▲^Clinically feasible

Although there is ample evidence that TAMs are preferentially M2-polarized (for instance, roughly 70% of TAMs are M2-like in non-small cell lung cancer [37]), the basis of the regulation and maintenance of this polarization imbalance remains unclear. In the TME, several factors can influence the macrophage phenotype. Cytokines such as TGFβ, IL10 and IL4; growth factors such as epidermal growth factor (EGF), macrophage colony-stimulating factor (M-CSF) and granulocyte-macrophage colony-stimulating factor (GM-CSF) and lipid mediators such as sphingosine-1-phosphate (S1P) and prostaglandin E2 (PGE2) promote a tumor-promoting phenotype [38–40]. However, mixed polarization phenotypes have been described in human ovarian carcinoma and pancreatic ductal adenocarcinoma [41, 42]. In ovarian carcinoma, the expression of the M2 marker CD163 on TAM surface correlates with patient relapse-free survival, although gene expression profiles reveal an unrelated M1/M2 mixed-polarization phenotype [42]. Additionally, CD163 expression correlates with the levels of IL6 and IL10, which exhibit context-dependent pro-inflammatory and/or anti-inflammatory functions [42]. Furthermore, freshly isolated TAMs from pancreatic ductal adenocarcinoma display M1 (HLA-DR, IL1B, TNFα) and M2 (CD163, IL10) characteristics [41]. A mixed phenotype is also evident at the transcriptional level, where differential expression of *STAT1* and *STAT3* lead to gene expression profile that cannot be categorized exclusively as M1 or M2 [34]. Furthermore, TAM heterogeneity also depends on their localization. Perivascular migratory TAMs are CD68^hi^MHCII^hi^CD206^low^ and have a more M1-like profile. Sessile TAMs resemble a more M2-like or “trophic” phenotype, which are CD68^hi^MHCII^low^CD206^hi^ and are mainly found at the tumor–stroma border and in hypoxic regions within the tumor mass [38, 43]. Indeed, solid tumors contain areas of hypoxia that triggers increased accumulation of macrophages and leads to upregulation of HIF-1α and HIF-2α, which enhance HIF-mediated expression such as VEGF and the glucose receptor GLUT1 in TAMs, to contribute to tumor angiogenesis and sustains tumor progression [44]. Also, the stability of HIF-1α and HIF-2α is controlled by PTEN/PI3K/AKT signaling axis - expression of PTEN and inhibition of PI3K/AKT signaling induces the degradation of HIF-1α and HIF-2α in a proteasome-dependent manner in TAMs [44]. Additionally, the localization of TAMs in hypoxic niches is controlled by a Sema3A/Neuropilin-1 signaling axis, which elicits PlexinA1/PlexinA4-mediated stop signals that maintain TAMs in hypoxic area [45]. And tumor hypoxia selectively promotes M2 macrophage polarization by activating ERK signaling triggered by IL6 in Lewis lung carcinoma [46]. Therefore, hypoxia is crucial for maintaining the M2-like pro-malignancy phenotype of TAMs and targeting hypoxia-mediated polarization of TAMs might be a practical strategy for cancer treatment.

### Significance and mechanisms of TAMs in tumor progression

Different mechanisms govern tumor initiation and progression promoted by TAMs. Macrophages contribute cancer-initiating inflammatory responses because expression of the anti-inflammatory transcription factor STAT3 is inhibited. Genetically inactivating *Stat3* in macrophages gives rise to chronic inflammation in the colon which creates a mutagenic microenvironment and subsequently causes invasive carcinoma [47]. Moreover, STAT3 is a critical maintainer of cancer stem-like cells (CSC), and M2-like TAMs secret activators of STAT3 such as oncostatin M and IL10 to promote tumor cell activation and proliferation via interaction between TAMs and tumor cells [9]. Although accumulating evidence suggests an anti-tumor role of M1-like TAMs [36, 48], more studies are required to clearly demonstrate whether macrophages in a cancer-initiating inflammatory environment are capable of eliminating cells that undergo aberrant transformation. Additionally, TAMs support tumor development by interacting with T cells. M2-like TAMs either produce immunosuppressive factors such as IL10 and TGFβ to inhibit CD4^+^ and CD8^+^ T cell effector function or secret chemoattractant such as CCL3, CCL4, CCL5, CCL18 and CCL22 to recruit factors associated with Tregs by targeting chemokine receptors CCR4, CCR5, CCR6 and CCR10 to TME to suppress the anti-tumor response [8].

Moreover, TAMs also play a pivotal role in tumor metastasis. VEGF as well as type IV collagenases MMP2 and MMP9 produced by M2-like TAMs not only promote tumor growth and angiogenesis, but also cause vascular permeability to facilitate tumor migration [10]. Therefore, TAMs contribute to both intravasation and extravasation. Because recruitment of TAMs to target vessels to induce vascular permeability requires CCL2 and colony-stimulating factor 1 (CSF1) synthesized by tumor cells to target receptor CCR2 and CSF1R on TAMs [4, 49], inhibition of CCR2 or CSF1R signaling reduces tumor growth and metastasis [50–53]. Moreover, TLR4 on TAMs can be targeted by serum amyloid A3 to promote metastasis through establishing premetastatic niches that constitute ‘homing signals’ to provide an environment to guide tumor cell adhesion and invasion [54]. Additionally, EMT is a key step for invasiveness and metastasis of tumor cells and recruitment of TAMs to the tumor site promotes tumor progression by enhancing EMT. Activation of TLR4 on M2-like TAMs elevates IL10 production and promotes EMT in pancreatic cancer cells [55]. Additionally, M2-like TAMs secrete EGF-like ligands to activate EGFR pathway in lung cancer cells, which ultimately promoting EMT that can be inhibited by a cannabinoid receptor 2 (CB2) agonist JWH-015 via downregulation of EGFR signaling [11]. Thus, regulation of metastasis-promoting M2-like TAMs is a rational method to inhibit tumor metastasis and progression.

## TAM-TARGETED IMMUNOTHERAPY

Aside from conventional therapies, immunotherapy has emerged as an effective strategy for cancer treatment. Vaccination with tumor antigens, adoptive cellular therapy with *in vitro* activated T cells and NK cells, and oncolytic viruses are approaches of immunotherapies to activate effector immune cells. The most promising strategy, which is scheduled to begin clinical application, is administration of antibodies against immune-checkpoint molecules such as CTLA-4, PD1 and ligand its PDL1 to neutralize immunosuppression [56]. Clinical evidence shows that an increased number of M2-like TAMs correlates with treatment failure and poor prognosis in different cancers types. And M2-like TAMs express not only ligand for CTLA-4 but also PDL1, thereby contributing immunosuppressive activity and providing target for therapy with anti-PDL1 [57]. Therefore, TAM-targeting immunotherapies represent a promising cancer therapeutic approach. These immunotherapeutic strategies include interference with TAM survival, repression of macrophage recruitment and repolarization of tumor-promoting M2-like TAMs towards tumor-suppressive M1-like TAMs (Table 1).

### Interference with TAM survival

Inducing apoptosis of TAMs appears to be an effective immunotherapeutic tactic for tumors. Trabectedin (ET-743) is an anti-tumor agent that, with respect to immune cells, is specifically cytotoxic to mononuclear phagocytes. The specificity is due to activation of caspase-8, which is essential for monocyte apoptosis via Fas and TNF-related apoptosis inducing ligand receptors (TRAILRs), whereas neutrophils and T cells are protected from depletion by the presence of a decoy receptor [58]. In addition, liposomal bisphosphonates, which can be phagocytized by macrophages, are widely considered as a promising drug for macrophage ablation. For instance, administration of liposome-encapsulated bisphosphonate clodronate leads to depletion of macrophages and reduces tumor progression [59]. Compared with clodronate liposomes, liposomal trabectedin targets all macrophage subsets in tumors to a similar extent but leads to more persistent macrophage depletion. Mechanistically, trabectedin upregulates TRAIL-R2 and Fas-associated protein with death domain (FADD) that facilitate the recruitment of caspase-8 and the activation apoptotic cascade in macrophages [58]. However, targeting all subtypes of macrophages is not an ideal way to deplete M2-like TAMs. And the issue of introducing specific agents that are more specific to M2-like TAMs might be addressed by a peptide (M2pep) with high affinity for murine M2 macrophages, thereby selectively abrogating M2-like TAMs and consequently improving the survival rate of tumor-bearing mice [60].

Furthermore, targeting cell surface proteins that are highly expressed in M2-like TAMs is a practical approach to reducing TAM survival. Legumain is an ideal target because it is highly expressed in M2-like TAMs in murine breast tumor tissues, whereas M1-like TAMs do not express legumain. A legumain-based DNA vaccine stimulates CD8^+^ T cells and selectively abrogates M2-like TAMs in mice with metastatic breast, colon and lung cancers, thereby increasing survival rate and regression of metastasis and angiogenesis [13]. Scavenger receptor A (CD204), which is highly and specifically expressed on the surface of M2-like TAMs, is also a promising target. Administration of anti-CD204 immunotoxin to mice challenged with peritoneal ovarian cancer eliminates TAMs and impedes tumor progression [61]. An RNA aptamer that targets murine or human IL4Rα/CD124 on TAMs can also promote TAM apoptosis with increasing CD8^+^ T cell infiltration *in vivo* [14]. Puig-Kröger et al. identified folate receptor β as a marker for M2-like TAMs, and targeting this protein using a recombinant immunotoxin in mouse glioma xenografts dramatically abrogates TAMs and suppresses tumor growth [62, 63]. Although it is unclear whether depletion of TAMs alone is effective for eliminating human cancer, targeted abrogation of TAMs in conjunction with anti-tumor agents may improve cancer therapy.

### Inhibition of macrophage recruitment

Tumor-derived chemokines, including CCL2 and CSF1, recruit peripheral monocytes to the tumor site [4]. Within the TME, peripheral monocytes differentiate into tumor-suppressive M1-like or tumor-promoting M2-like subsets in response to distinct microenvironmental signals that are specific to each tumor stage. Therefore, targeting these signaling molecules is another potential strategy to inhibit the accumulation of TAMs.

CCL2 is highly produced by bone marrow osteoblasts, endothelial cells and stromal cells as well as tumor cells, including breast cancer, prostate cancer and myeloma cells [49, 50, 52]. CCL2 plays pivotal roles in tumorigenesis and metastasis, especially bone-targeted metastasis, by both directly promoting tumor cell proliferation, migration and acting as a chemotactic factor to recruit macrophages that express the CCL2 receptor CCR2 to the tumor site, inducing an inflammatory response that promotes tumor growth [52]. Blockade of either CCL2 or CCR2 has shown pre-clinical anti-tumor success. The CCL2 inhibitor bindarit significantly suppresses M2 macrophage recruitment and tumor growth in human melanoma xenografts [50]. Additionally, neutralizing antibodies against CCL2 (anti-human CNTO888 and anti-mouse C1142) in combination with docetaxel diminish prostate cancer cell–mediated tumor burden and induce tumor regression [51]. Moreover, applying the CCR2 kinase antagonist PF-04136309 to murine pancreatic cancer inhibits M2 macrophage recruitment and reduces cancer progression [52, 64].

CSF1 and its receptor CSF1R regulate macrophage homeostasis by modulating their proliferation, differentiation and migration. Blockade of the CSF1/CSF1R axis by inhibitors and/or neutralizing antibodies efficiently decreases macrophage recruitment. For instance, each of the CSF1R inhibitors PLX6134, GW2580 and PLX3397 reduces M2 macrophage infiltration and improves chemotherapeutic efficacy with enhanced CD8^+^ T cell responses [64]. Additionally, inhibition of CSF1 using either an antisense oligonucleotide or anti-CSF1 antibody suppresses macrophage recruitment and results in reduced tumor growth in human MCF-7 breast cancer cell–xenografted mice [65]. From a mechanistic perspective, MMP2, MMP12 and VEGFA, which are produced by macrophages and are important in tumor invasion and angiogenesis, are downregulated upon blockade of the CSF1/ CSF1R axis [65, 66]. Likewise, the monoclonal antibody (mAb) RG7155 against CSF1R reduces macrophage infiltration and enhances CD8^+^ T cell responses in diffuse-type giant cell tumors [67]. In addition to mAbs and inhibitors, a study on hepatocellular carcinoma showed that miR-26a expression downregulates CSF1 and leads to inhibition of TAM recruitment [68]. A recent study showed Luteolin that is a common flavonoid derived from various herbal plants suppresses STAT6 activation and CCL2 secretion triggered by IL4 in TAMs, which leads to reduced recruitment of macrophages to tumors as well as decreased migration of Lewis lung carcinoma cells [69]. Apart from decreasing accumulation of TAMs, targeting CSF1/ CSF1R axis is also capable of repolarizing M2-like TAMs to an M1-like phenotype. For instance, in a mouse proneural glioblastoma multiforme model, the CSF1R inhibitor BLZ945 targets TAMs and leads to reduced M2-associated genes such as *arginase 1* and *CD206*, but BLZ945 does not affect the number of TAMs [53].

Interestingly, Wang and Kubes recently proposed a non-vascular route for peritoneal macrophage recruitment, which they referred to as “wormhole migration”, [70]. In this novel paradigm, fully differentiated GATA-binding protein 6^+^ macrophages are recruited from the peritoneal cavity to the liver through the mesothelium. However, whether tumor cells similarly induce peritoneal macrophage recruitment and whether this non-vascular macrophage migration can be targeted as a cancer therapeutic strategy require further study.

### Repolarization of M2-like TAMs towards an M1-like phenotype

As mentioned above, macrophages are functionally plastic because they are induced in response to and modulated by the alteration of molecules in the TME, including cytokines, chemokines, pattern recognition receptors and hormones [32, 33]. Therefore, manipulation of environmental stimuli to repolarize M2-like TAMs to a tumor-suppressive phenotype under pathological conditions is a potential clinical strategy for cancer therapy. Administration of IL12 to mice bearing hepatocellular carcinoma cell–based tumors alters the functional phenotype of M2-like TAMs by downregulation of Stat3 and its downstream transcription factor c-myc, thereby reducing the production of tumor-promoting cytokines and inhibiting tumor growth [71]. In addition, TAMs derived from human ovarian cancer ascites are repolarized to an M1-like phenotype, producing less CCL18, MMP9 and VEGF, by being exposed to IFN-γ [36]. Furthermore, injection of polyinosinic:polycytidylic acid (polyI:C) into Lewis lung carcinoma tumor–implanted mice to activate the TLR3/Toll–IL1 receptor domain–containing adaptor molecule 1 (TICAM-1) switches tumor-promoting macrophages into tumor suppressors [72]. Intriguingly, apart from cytokine therapy to modify the immunosuppressive microenvironment by boosting T cell–based anti-tumor activity, bacteria-mediated tumor therapy has been shown to be a promising strategy [73]. For instance, introduction of attenuated *Listeria monocytogenes* to the TME of ovarian cancer–bearing mice switches M2-like TAMs into a tumoricidal phenotype and induces tumor cell lysis through Nos2-dependent production of nitric oxide [48]. Bacillus Calmette-Guérin (BCG) vaccine directed against *Mycobacterium bovis* has also been applied to treat bladder cancer because it enhances the cytotoxic potential of macrophages [74]. Similarly, a recent study showed that heat-killed *Mycobacterium indicus pranii* induces repolarization of TAMs derived from B16F10 tumors towards a tumor-suppressive M1-like phenotype [75].

Additionally, abnormal tumor vasculature, which can be caused by M2-like TAMs, is one of the key hallmarks of cancer. Abnormal tumor vasculature has detrimental effects on tumor progression because it changes the TME and promotes metastasis. Therefore, vascular normalization is considered as a potential approach for improving anti-tumor therapy. The anti-angiogenic effect of zoledronic acid, a clinical agent for inhibition of spontaneous mammary carcinogenesis, is partly due to repolarization of pro-angiogenic M2-like TAMs to suppressive M1-like TAMs [76]. However, the mechanism of zoledronic acid–induced repolarization has not yet been deciphered. Histidine-rich glycoprotein repolarizes M2-like TAMs to enhance anti-tumor immune responses and vessel normalization via downregulation of placental growth factor (PlGF) [77]. Likewise, the STAT3 phosphorylation inhibitor hydrazinocurcumin converts TAMs to an M1-like phenotype to suppress angiogenesis and metastasis in breast cancer [78]. And 5,6-dimethylxanthenone-4-acetic acid (DMXAA) repolarizes M2-like TAMs towards an M1-like phenotype which has an effect on mediating the vascular disrupting via STING activation in mouse models of non-small-cell lung cancer [79].

The pro-inflammatory transcription factor NF-κB is inactivated by binding to its inhibitor IκB in the cytoplasm. The activation of TLRs and the receptors for IL1 and TNFα, which triggers the phosphorylation and subsequentdegradation of IκB, activates NF-κB and allows its translocation into the nucleus where it promotes transcription of several genes encoding cytokines and immune effectors, thereby initiating a robust pro-inflammatory response [80]. TLR agonists, such as polyI:C, CpG-oligodeoxynucleotide (CpG-ODN), TLR9 ligand and anti-IL10R, switch M2-like TAMs into M1-like cells with enhanced anti-tumor immunity [72, 81]. Pseudomonas aeruginosa strain (PA-MSHA) induces TAMs to undergo an M1-like polarization upon activation of NF-κB–induced expression of genes, which results in the inhibition of gastric carcinoma progression in mice [82]. However, the exact role of PA-MSHA in TAM repolarization has not yet been elucidated. In addition, another NF-κB pathway, which is initiated by ligands such as RANKL, LTα, LTβ and LIGHT produced by TAMs, targets tumor-promoting genes like CXCL12 and VEGFC via receptors such as LTβR, CD40 and RANK [80]. This non-canonical pathway is the molecular basis for anti-CD40–induced repolarization of TAMs from a tumor-promoting M2 to a suppressive M1-like phenotype with upregulation of IL12, TNFα and INF-γ in KPC mice which spontaneously develop pancreatic ductal adenocarcinoma [83]. Restraining immunosuppression and tumor-promoting activities within the TME is a fundamental goal of immunotherapy for cancer. Fujiwara et al. screened approximately 200 natural compounds and found that corosolic acid suppresses macrophage differentiation into M2-like cells by suppressing the activities of both STAT3 and NF-κB [84]. In a murine sarcoma model, administration of corosolic acid significantly impedes subcutaneous tumor development and lung metastasis by reversing immunosuppressive function of M2-like TAMs and increasing CD8^+^ T cell infiltration [12].

Aside from the NF-κB pathway, the MAPK/ERK pathway is also involved in M2-like TAM repolarization. The small molecule compound copper *N*-(2-hydroxy acetophenone) glycinate (CuNG) triggers the formation of reactive oxygen species followed by p38 MAPK and ERK1/2 activation and intracellular glutathione upregulation. Activation of p38 MAPK then leads to IL12 production, and ERK1/2 enhances the generation of IFN-γ which in turn prolongs IL12 production and downregulates TGFβ. Consequently, CuNG repolarizes M2-like TAMs towards a pro-immunogenic type [85].

Recently, microRNAs have emerged as novel molecular regulators of numerous genes and pathways involved in normal immune responses, in the pathogenesis of cancers, and inflammatory and autoimmune diseases [86]. Macrophages produce several active microRNAs, namely miR-125, miR-155 and miR-378, which are upregulated in M1 macrophages, and M2 macrophages express miR-9, miR-21, miR-146, miR-147, miR-187 and miR-511-3p [86]. In particular, overexpression of miR-155 repolarizes M2-like TAMs to M1 macrophages, whereas depletion of miR-155 impedes M1 macrophage polarization induced by LPS and IFN-γ [87]. Mechanistically, miR-155 contributes to maintaining the M1-like phenotype by both enhancing pro-inflammatory signaling and attenuating alternative activation and is regulated by NF-κB in response to TLR ligands and IFNs in macrophages. In addition, miR-155 enhances TNFα expression while reducing the production of inhibitors of the pro-inflammatory response, including IL13 and (C/EBP)β [86, 87]. The intronic microRNA miR-511-3p is encoded by *Mrc1* that encodes M2 marker CD206. Although miR-511-3p is upregulated in M2 macrophages, experimentally induced upregulation of miR-511-3p attenuates the tumor-promoting functions of M2-like TAMs by targeting *Rock2*, which promotes the M2-like phenotype of macrophages by enhancing expression and secretion of ECM that facilitate tumor growth and vascularization [88]. However, the detailed mechanisms underlying strand selection by miR-511-3p and significance of M2-like TAM repolarization remain to be investigated. In contrast, a study determined that TRAIL repolarizes M2-like TAMs to display anti-tumor properties associated with negative regulation of miR-146a, which leads to a heightened inflammatory response in a time- and dose-dependent manner [89]. This study demonstrated that histone deacetylases 1 (HDAC1) contributes to the negative regulation of miR-146a expression at the transcriptional level in TRAIL-stimulated macrophages [89]. In addition to HDAC1, other epigenetic modifiers such as HDAC3 and histone demethylase Jmjd3 are also involved in regulating macrophages heterogeneity and functions [90, 91]. However, further research is warranted to evaluate the roles of other HDACs and JMJDs in macrophage polarization and determine whether regulation of these epigenetic modifiers can repolarize M2-like TAMs to tumoricidal effectors.

## IMPROVING THE TARGETED DELIVERY OF DRUGS TO MACROPHAGES

It has been an ongoing challenge to transport drugs to specific cell types during cancer treatment. Systems to deliver liposomes, nanoparticles and microspheres have been developed to enhance drug efficacy. Nanoparticles are in the 1- to 100-nm size range, whereas liposome diameters vary from 400 to 2500 nm [92]. Macrophages are professional phagocytes and thus have superior capacity to engulf nanoparticles and liposomes. Consequently, nanoparticle and liposome formulations have been developed to transport anti-tumor drugs by TAMs with high specificity and low toxicity to the organism. Nanoparticles are used in different formulations ranging from solid lipid nanoparticles (SLNs) to polymer-, gold- or albumin-based nanoparticles. To date, several nanoparticle formulations have shown clinical feasibility, including solid lipid nanoparticles loaded with the topoisomerase inhibitor mitoxantrone, polymer nanoparticles loaded with the anti-tumor agents cisplatin and cyclodextrin and the albumin nanoparticle–based Abraxane [92, 93]. Liposomes contain a phospholipid bilayer to which additional molecules can easily be added, and small liposomes (50–100 nm) liposomes that have been negatively charged by introducing negatively charged lipids such as phosphatidylserine and phosphatidylglycerol are preferably engulfed by macrophages [94]. In addition, it has been observed that ligand-containing liposomes are more efficiently engulfed than those without ligand [95]. Specifically, liposomes conjugated with a peptide (GGPNLTGRW or RGD) selectively target integrin receptors of monocytes [96]. Liposomes coated with antibodies (immunoliposomes) are able to bind to the Fc receptors of macrophages. For example, CD163 antibody–coated liposomes can be used to target M2 macrophages [97]. Moreover, mannosylated liposomes, which target lectin receptors of macrophages and DCs, have been developed to transport anti-tumor agents such as CpG-ODN and DNA [98]. Liposomal Doxil and abovementioned liposomal clodronate are successful examples of a liposome-based cancer treatment with low toxicity and high specificity [59, 92]. Although liposome-mediated depletion of TAMs has been demonstrated, whether liposome-encapsulated agents can effectively facilitate M2-like TAM repolarization still requires further investigation.

## CONCLUSIONS AND FUTURE PERSPECTIVES

TAMs play dual roles in tumor growth. They have anti-tumor features in the early stages of tumors, whereas with tumor progression, TAMs adopt a tumor-promoting M2-like phenotype characterized by activation of Th2 signaling in the TME. Thus monocytes are recruited to tumor sites in response to factors secreted by tumor cells and transformed into M2-like TAMs to facilitate invasiveness of tumor cells in more advanced stages. In addition, experimental and clinical studies have revealed that greater infiltration by TAMs correlates with worse patient outcomes. Anti-tumor therapeutic strategies targeting TAMs include lowering TAM survival, reducing macrophage recruitment and switching M2-like TAMs into an M1-like phenotype. Among these strategies, reverting M2-like TAMs to the tumor-suppressive phenotype by modulating the TME ismost promising one because phenotypes of macrophages are highly sensitive to stimuli within the TME. Of note, promoting the generation of M1 macrophages from monocytes also can be a feasible method for accumulating of tumoricidal effectors at tumor sites to slow progression of the cancer. Although increasing the circulating level of monocyte chemoattractant protein-1 (MCP-1) to a threshold level enhances the anti-tumor effects of suicide gene therapy against hepatocellular carcinoma via M1 macrophage activation, it is unclear whether M1 activation is due to M2-like TAM repolarization or promotion of monocytes to differentiate to M1 macrophages because monocyte recruitment depends on the level of MCP-1 secreted by tumor cells [99]. Therefore, further investigations are required to identify more effective approaches to elevate ratio of M1-like to M2-like TAMs to prevent tumor progression and recurrence. Several therapeutic drugs targeting TAMs are currently available for clinical use. For instance, the agent trabectedin lowers TAM survival [58] and alemtuzumab eliminates TAMs by targeting a TAM surface protein [100]. However, the efficacy of such cancer therapy must be improved via the development of additional agents that are more specific to TAMs and less cytotoxic.

Although a 100% efficient receptor blockade, as could be achieved with a genetic knockout in mice, is unlikely to achieve the general pharmacodynamic and kinetic properties of xenobiotics, it would be useful for identifying key differentiators of the M2 macrophage lineage. To target TAMs more effectively, we must identify key differentiators of the M2 macrophage lineage or monocyte to M1 macrophage lineage. Furthermore, more *in vivo* studies are required to evaluate the toxicity and efficacy of nanoparticles and liposome-based cancer treatment.

Investigations of genetic and epigenetic mechanisms of macrophage heterogeneity and polarization will establish a foundation for macrophage phenotype reversion strategies. Owing to the diversity of macrophages within the TME, more macrophage markers (especially function-related) that are specific to individual macrophage subsets need to be identified to facilitate a better understanding of the mechanisms of spatiotemporal modulation of macrophage polarization and repolarization. In addition, because TAM infiltration is associated with poor patient outcomes, systematic and well-defined criteria for the evaluation of macrophage populations are required for practical TAM-targeting diagnostic and therapeutic strategies.
